# Enhancing Localized Evaporation through Separated Light Absorbing Centers and Scattering Centers

**DOI:** 10.1038/srep17276

**Published:** 2015-11-26

**Authors:** Dengwu Zhao, Haoze Duan, Shengtao Yu, Yao Zhang, Jiaqing He, Xiaojun Quan, Peng Tao, Wen Shang, Jianbo Wu, Chengyi Song, Tao Deng

**Affiliations:** 1 State Key Laboratory of Metal Matrix Composites, School of Materials Science and Engineering, Shanghai Jiao Tong University, 800 Dong Chuan Road, Shanghai 200240, P.R.China; 2 MOE Key Laboratory for Power Machinery and Engineering, School of Mechanical Engineering, Shanghai Jiao Tong University, 800 Dong Chuan Road, Shanghai 200240, P.R.China

## Abstract

This report investigates the enhancement of localized evaporation via separated light absorbing particles (plasmonic absorbers) and scattering particles (polystyrene nanoparticles). Evaporation has been considered as one of the most important phase-change processes in modern industries. To improve the efficiency of evaporation, one of the most feasible methods is to localize heat at the top water layer rather than heating the bulk water. In this work, the mixture of purely light absorptive plasmonic nanostructures such as gold nanoparticles and purely scattering particles (polystyrene nanoparticles) are employed to confine the incident light at the top of the solution and convert light to heat. Different concentrations of both the light absorbing centers and the light scattering centers were evaluated and the evaporation performance can be largely enhanced with the balance between absorbing centers and scattering centers. The findings in this study not only provide a new way to improve evaporation efficiency in plasmonic particle-based solution, but also shed lights on the design of new solar-driven localized evaporation systems.

This paper studies localized evaporation of solution with the mixture of light absorptive nanoparticles (plasmonic absorber) and light scattering nanoparticles (polystyrene nanoparticles (PSNPs)). Evaporation involving liquid-to-gas phase change has been recognized as one of the key energy conversion processes to be utilized in modern industries^1^. Plasmonic effect in nanostructures, induced by light-driven collective oscillations of charge carriers, has attracted tremendous attention due to its unique optical, electrochemical and photothermal properties^2^,^3^,^4^,^5^,^6^,^7^,^8^. Recently it was reported that, under light illumination, plasmonic nanoparticles suspended in water were able to convert light to heat and generate water vapor with fairly high evaporation efficiency (i.e. the ratio of the energy used for the vaporization of water to the total energy input)^9^,^10^,^11^,^12^. As shown in Fig. 1a, when light propagates through a solution containing gold nanoparticles (AuNPs), each AuNP converts input optical energy into thermal energy and the resulting energy heats up the surrounding water. Vapor bubbles are continuously generated within the bulk water and the vapor inside bubbles can be released after they travel a long distance to reach the top surface of water^13^,^14^,^15^. In such process the heat transfer between freshly generated bubbles and surrounding bulk water is unavoidable and eventually large portion of energy is used to heat the bulk water rather than generating steam^16^.

Significant efforts have been directed toward improving the evaporation efficiency of aqueous plasmonic nanoparticle-based system. Modulating the structural metrics of light absorbers by changing the shape or composition of nanoparticles leads to the increased absorption cross section or broadband absorption^17^,^18^,^19^,^20^,^21^,^22^,^23^. Nevertheless, heating bulk water still exists in the plasmonic nanoparticle-based evaporation system when the light is absorbed deep into bulk solution. To minimize the heat loss of currently existing evaporation system, localizing generated heat within the top water layer is a feasible way to avoid heating bulk water and to improve the evaporation efficiency^16^,^24^,^25^,^26^. For example, Halas *et al.* have demonstrated such localization through solutions containing composite plasmonic nanoparticles with both scattering and absorption properties, which help localize incident light on the surface of solution and enhance light-to-heat conversion^24^. They reasoned that the multiple light-scattering from the composite particles assists in decreasing the penetration depth, resulting in the absorption of most of incident light in a short distance. The generated heat hence is localized at the top surface of water and the evaporation efficiency is improved. In their system, same composite particles served as both the light scatterers and light absorbers. While improved performance has been demonstrated, the competing nature of light interaction on the same particle might intrinsically limit the tunability and optimization of such localized evaporation systems.

In this paper, rather than using the same nanoparticles that are both the light scattering centers and the light absorbing centers, we developed an alternative approach that separated these two functions and used mixtures of nanoparticles with primarily light absorbing function and the nanoparticles with primarily light scattering function to achieve the localization (Fig. 1b). Such system with separated functions enabled us to independently tune the light absorption and light scattering to optimize the performance of the localized evaporation system. In this study, we used 10-nm AuNPs as pure light absorbers and found that concentrated 10-nm AuNPs (aq) had performed better in evaporation efficiency than AuNPs with sizes larger than 10 nm. As the concentration of 10-nm AuNPs increases, light absorption is localized close to the top surface of water, implying that solution with high density of 10-nm AuNPs would shorten the path length of incident light, which leads to localized heating of the top small portion of bulk water. Such a highly concentrated solution, however, has low efficiency in material usage since most of AuNPs at the bottom of the concentrated solution make no contribution to evaporation. To improve the material usage and also at the same time achieving localized evaporation, we studied the solution with mixed particles composed of pure light absorbers (10-nm AuNPs) and pure light scattering particles (PSNPs) to lower the usage of AuNPs. With the mixing of PSNPs, effective multi-scattering is induced at the top portion of the solution and the incident light could be scattered in all directions and reabsorbed by AuNPs. The evaporation efficiency has shown an enhancement as high as ~58.9% after adding PSNPs (6.07 × 10^10^ particles/ml) into solution of 10-nm AuNPs (3.59 × 10^11^ particles/ml). With the demonstrated absorption and scattering behavior of solution of AuNPs/PSNPs and added understanding of performance enhancement through scattering-based light trapping, this study provides new insight in designing efficient evaporation system and also possibility to tune the evaporation performance of such systems.

## Results

**Figure 2:**
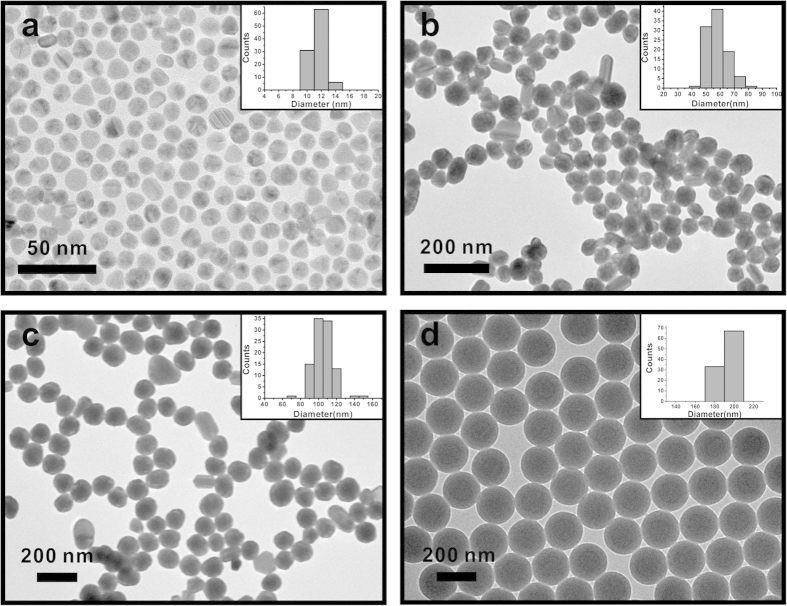
TEM images of (**a**) 10-nm AuNPs (11.50 ± 1.04 nm); (**b**) 50-nm AuNPs (57.54 ± 7.32 nm); (**c**) 100-nm AuNPs (105.14 ± 12.01 nm); (**d**) 200-nm PSNPs (192.62 ± 4.26nm).The insets in the images show the size analysis of the particles.

**Figure 3:**
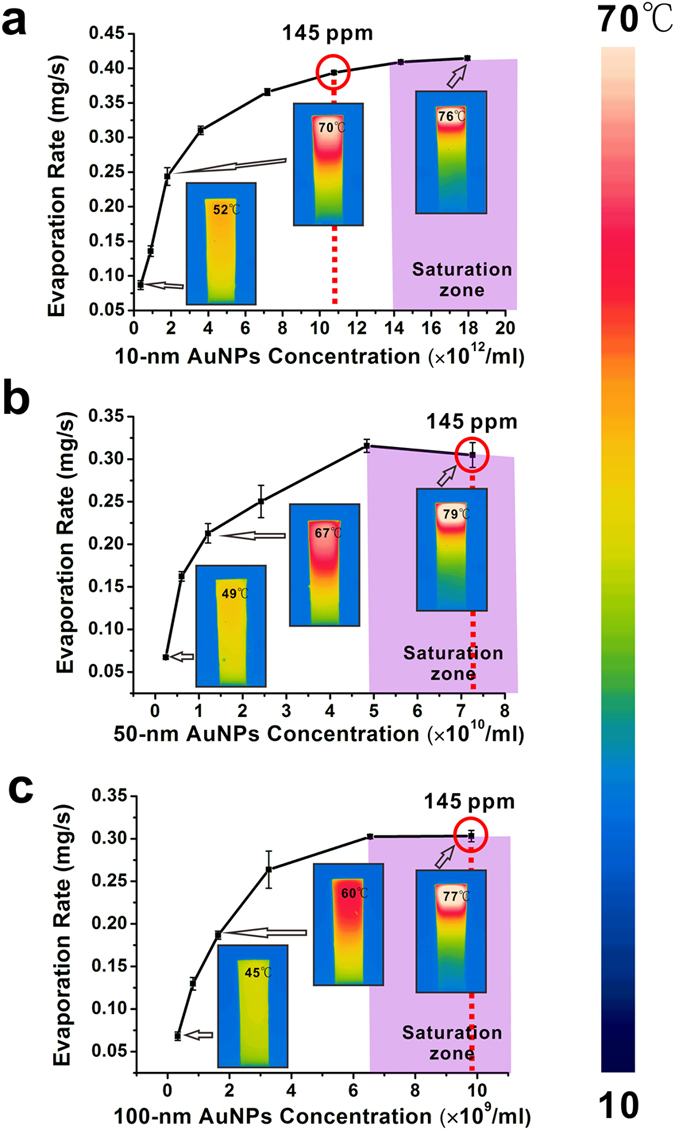
Evaporation performance of 10-nm (**a**) 50-nm (**b**) 100-nm (**c**) aqueous AuNP solution with different concentration under the illumination of 532-nm laser light with the power density of 35.36 W/cm^2^. (The insets are thermal mapping images taken from IR camera).

Solutions of AuNPs of various sizes were fabricated via seed-mediated growth method^27^. Fig. 2a–c shows the synthesized AuNPs with expected diameters (see Methods for synthetic details). To characterize the performance of AuNP-based evaporation system, evaporation rates have been plotted as a function of the concentration of AuNPs with different sizes under the same 532-nm green laser illumination with the power density of 35.36 W/cm^2^. The AuNPs were stabilized by citrate, and were relatively stable with no noticeable agglomeration during the evaporation experiments (Table S1).As shown in Fig. 3, the evaporation rate of 10-nm AuNP solution rises steeply at the initial stage and reaches its steady state or plateau after its concentration falls on the saturation zone. Figure 3b,c also show similar behavior for the 50-nm and 100-nm AuNP solution. We attributed this saturation mostly to the intense light absorption of highly concentrated AuNPs at the top portion of solution and the plasmonic-induced heat is localized. To further support our hypothesis, IR thermal mappings are included in the same plot to show the temperature distribution within AuNP solutions. As shown in the inserted IR images of Fig. 3a, the temperature is uniformly distributed over the most diluted 10-nm AuNP solution and shows no sign of localization. On the contrary, after the concentration increased by thirty-fold and fell into the saturation zone, the surface temperature jumped by 24 °C and the bottom of the 10-nm AuNP solution remained cool. The thermal diffusion to bulk water was impeded and only a small portion of the overall volume of the solution was heated. The same case also occurs for the 50-nm and 100-nm AuNP solution (Fig. 3b,c).

Though the evaporation saturation behaviors of 10-nm, 50-nm and 100-nm AuNPs can resemble one another, the mechanisms of light absorption at the top water layer are intrinsically different. On the basis of Mie theory, the extinction of plasmonic nanoparticles is composed of absorption and scattering. When the nanoparticle size is relatively large, its scattering effect will become important and cannot be neglected^28^,^29^. For example, for the incident light with wavelength of 532 nm, though 100-nm AuNPs exhibits only a bit large absorption cross section of 2.14 × 10^−14^ m^2^ compared to that of 50-nm AuNP’s (1.19 × 10^−14^ m^2^), the scattering cross section of 100-nm AuNP increases by a factor of 8 compared to that of 50-nm AuNPs (50-nm AuNP: 3.09 × 10^−15^ m^2^; 100-nm AuNP: 2.52 × 10^−14^ m^2^). However, when the size of AuNPs is less than 10 nm, they exhibit no scattering effect according to Mie theory^29^. Hence, the multi-scattering effect dominates the evaporation rate saturation in 50-nm and 100-nm AuNP solution^24^ while it plays no role in the evaporation rate saturation of 10-nm AuNP solution.

To understand the temperature distribution of purely absorptive 10-nm AuNPs with different concentration, a simple Lambert-Beer model is employed to describe the light absorption phenomenon in the solution-based evaporation system. According to Lambert-Beer equation (1) 28:


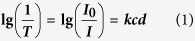


where ***T***is transmittance, 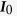
is incident light intensity,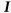 is transmission light intensity,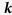 is absorption factor,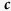 is molar concentration of absorptive nanoparticles, 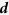 is path length of light. Under our experimental condition, the relationship between concentration and absorbance of 10-nm AuNP solution agrees closely with Lambert-Beer law (see the Methods for details)^30^. If we define a specific path length of light (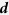), at which the absorption reaches 99% (***T*** = 1%), as the penetration depth (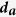), 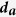 and 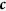 has the following relationship on the basis of equation (2):


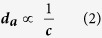


Under light illumination, 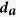 could be largely suppressed by increasing the concentration of particles to achieve highly localized heating and thus the confined high temperature region. More detailed calculation of 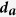 can be found in supporting information (Fig. S1)

Having understood the mechanisms of evaporation rate saturation of different sized AuNPs, comparison of mass-based photothermal conversion among 10-nm, 50-nm and 100-nm AuNPs (aq) was performed to determine which particles enhance evaporation rates most. To quantify the evaporation performance, we used the following equation (3) to calculate evaporation efficiency (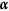):


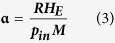


where ***R*** is evaporation rate, 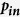 is input energy power,***M*** is molar weight of water, 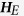
is molar heat of evaporation of water. The equation parameters are taken from experimental data shown in Fig. 3. The evaporation rates of 10-nm, 50-nm, and 100-nm AuNP solution of the same mass concentration (145 ppm) are 0.3937 mg/s, 0.30484 mg/s and 0.3031 mg/s, respectively, and the corresponding evaporation efficiencies 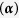 are 35.6%, 27.6% and 27.4%, respectively. Clearly, pure light absorbers (10-nm AuNPs) exhibit the highest evaporation efficiency among different-sized AuNPs. As the concentration increased, the solution of 50-nm and 100-nm AuNPs not only localized heat at the top portion of the solution, back scattering of incident light into air also increased in these solutions^31^, which resulted in the loss of part of incident light energy. For 10-nm AuNP solutions, light propagated directly through solution and attenuated exponentially without back scattering. Eventually, almost all of the incident light energy is transferred into heat within the solution containing 10-nm AuNPs. In the supporting information, we theoretically calculated the loss due to the reflection of normal incident light at the air-water interface (Table S2), the total reflectivity involving back scattering from nanoparticles (Fig. S1). Based on the calculated penetration depth (Fig. S1) and the reflection, we can calculate the absorbed power density (Fig. S2), which can help explain the trend of the evaporation rates observed for various solutions in the experiment.

## Discussion

Although highly concentrated 10-nm AuNP solution can work as an efficient light energy-harvesting system to maximize the light-trapping and confine the generated heat within the top portion of bulk water, most of the AuNPs have not been used to convert light to heat in this evaporation geometry. To reduce the amount of AuNPs used and at the same time to reach relatively efficient evaporation, purely scattering PS particles were mixed with *diluted* purely absorptive 10-nm AuNP particles in this work. A soap-free emulsion polymerization method was employed to synthesize PSNPs (~200 nm in diameter) with well-defined spherical morphology (Fig. 2d)^32^.

**Figure 4:**
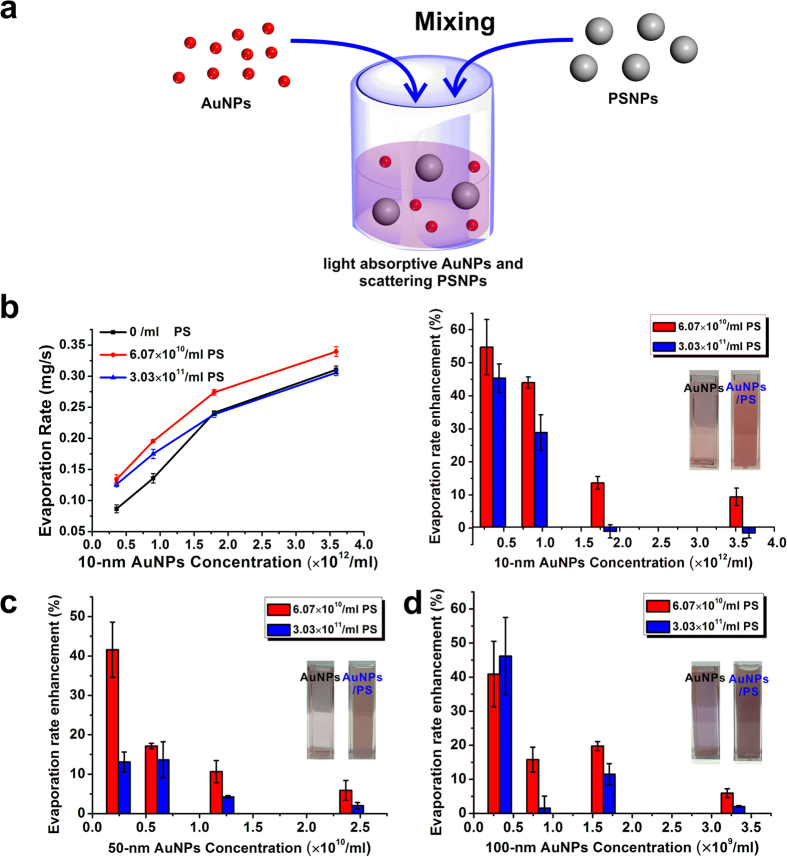
(**a**) Schematic illustration of PSNPs and AuNPs mixing process; (**b**) Left side: evaporation rate of 10-nm AuNPs and 200-nm PSNPs mixing solution as a function of AuNP concentration; right side: bar graph showing changes in evaporation rate due to adding of PSNPs (pure AuNP solution was used as standard); (**c**,**d**) Bar graphs for solutions containing 50-nm and 100-nm AuNPs. (Fig. 4a was drawn by Chengyi Song).

**Figure 5:**
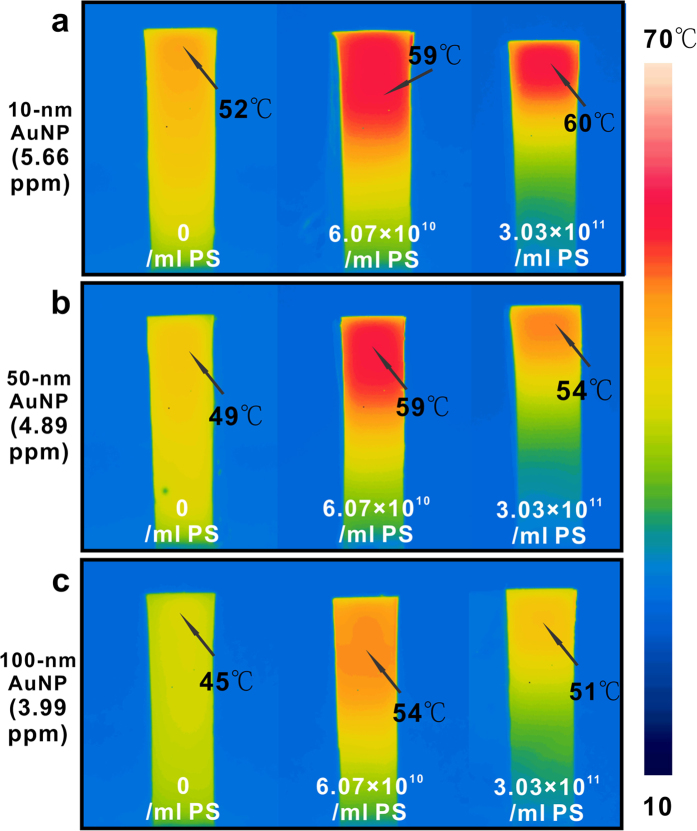
Temperature distribution of 10-nm (**a**), 50-nm (**b**) and 100-nm (**c**) AuNPs aqueous solution with or without mixing PSNPs under the illumination of 532-nm laser light with the power density of 35.36 W/cm^2^.

The solution with enhanced optical absorption was prepared by mixing AuNPs with PSNPs directly, which is shown in Fig. 4a. We examine the evaporation rate of the 10-nm AuNP solution as a function of concentrations of both PSNPs and AuNPs under the same input power of 35.37 W/cm^2^ (Fig. 4b). Addition of an aliquot of PSNPs (aq) into diluted AuNPs (aq) resulted in the enhanced water evaporation rate (see the left plot of Fig. 4b). To evaluate the enhancement performance of evaporation rate after adding PSNPs, we employ the following equation (4) to quantify the enhancement:


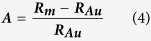


where ***A*** is evaporation rate enhancement percentage, 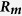
is the evaporation rate of mixed solution, 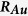 is the evaporation rate of pure AuNP solution. As shown by the red bar graphs of Fig. 4b, the evaporation rate enhancement percentage decreased from 54.7% to 9.4% when the concentration of 10-nm AuNPs (aq) was increased by a factor of 10. For the diluted solution of AuNPs, the scattering from the PSNPs helps maximize the light absorption at the surface close to the air-water interface, and such focused absorption increases the evaporation efficiency. For the concentrated AuNPs, most absorption already happens at the surface close to the air-water interface even without the scattering enhancement from PSNPs, so the evaporation rate enhancement decreases. Compared to the evaporation rate enhancement of 10-nm AuNPs after adding PSNPs, the evaporation rate enhancement of 50-nm or 100-nm AuNPs is lower than that of 10-nm AuNPs (Fig. 4c,d). Closely examining the IR images of 10-nm AuNP solution and the mixture solution of AuNPs and PSNPs under laser illumination, we found that the maximum temperatures remained constant and were relatively independent of the amount of PSNPs studied in our experiment (Fig. 5a). For 50-nm and 100-nm AuNPs, however, the temperatures reached their maximum after adding moderate amount of PSNPs and eventually decreased after excess amount of PSNPs was added (Fig. 5b,c). The problem is ascribed to which effect, *multi-scattering effect* or *back scattering effect at the water/air interface*, dominates. At initial stage, the PSNPs concentration is not high enough. Even though the back scattering induced by PSNPs occurs at the interface, most of light is absorbed by AuNPs and the generated heat is confined in a small volume, resulting in the increased temperature in the hot zone. As the PSNPs concentration is gradually increased, back scattering effect due to both of large-sized AuNPs and PSNPs will dominate. An unneglectable part of light is scattered back to the air at the interface, which leads to the decrease of temperature of the solution. Since 10-nm AuNPs show no sign of scattering and absorb light more efficiently than large-sized AuNPs, they make no contribution to the back scattering. Enhancing the concentration of PSNPs leads to the increased probability of inner multi-scattering and decreased light penetration depth at the top water layer, which alleviates the back surface scattering effect caused by the high concentration of PSNPs. Hence, the mixture of purely absorbed AuNPs and purely scattering PSNPs works more efficiently than the mixture of scattering AuNPs and PSNPs.

**Figure 6:**
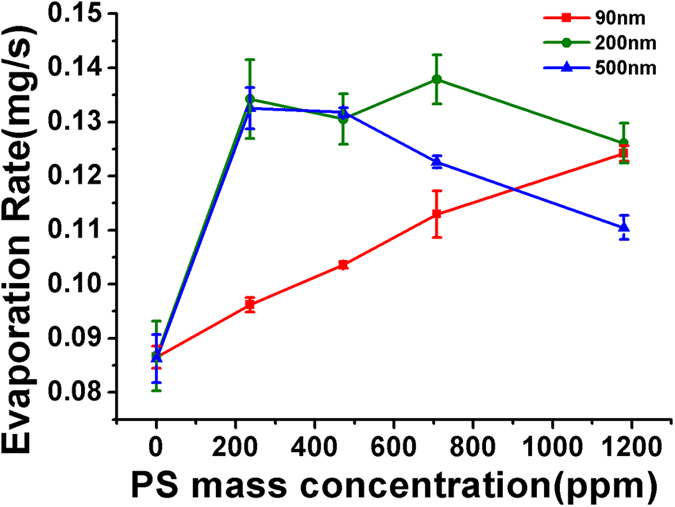
Evaporation rate changes as a function of PS mass concentration for solutions containing 10-nm AuNPs, and 90-nm, 200-nm or 500-nm PSNPs.

In this study, the evaporation performance of PSNPs of different sizes was also examined as a function of PSNP concentration. 236 ppm (mass concentration) of 500-nm PSNPs (Suzhou micro- and nano-technology Company, Suzhou, China) were mixed with exactly the same diluted solution of 10-nm AuNPs used in Figs 4 and 5. As shown in Fig. 6, the evaporation rate curve of the above solution reached its maximum and overlapped with diluted 10-nm AuNP solution containing 236 ppm of 200-nm PSNPs. As the PSNPs concentration increased further, the evaporation rate of the solution containing 500-nm PSNPs dropped quickly compared to 200-nm PSNPs. To elucidate this phenomenon, we introduce the scattering free mean path (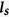) to evaluate the scattering effect of different-sized PSNPs (see Methods for derivation details). 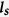 stands for the path length between two scattering events and a shorter 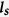 means stronger scattering effect. By fixing the mass concentration of 200-nm PSNPs and 500-nm PSNPs used in the experiment, we calculate the ratio of  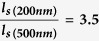, indicating that 500-nm PSNPs exhibit stronger scattering effect than 200-nm PSNPs. Especially, in concentrated 500-nm PSNP solution, most of light was scattered back to air at the interface, resulting in rapid drop in evaporation rate after it reached its maximum. The same mass controlled experiment was also conducted on 90-nm PSNPs (Aladdin industrial corporation, Shanghai, China). Such 90-nm PSNPs exhibit much weaker scattering effect than 200-nm PSNPs (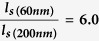). The evaporation enhancement for such small PSNPs is thus less than 200-nm PSNPs. The back scattering effect, however, is also smaller than 200-nm PSNPs, so there is no observed decrease in evaporation rate even at the concentration of 1200 ppm.

In this paper, we have developed a localized evaporation approach via separated light-absorbing centers and light scattering centers in the same evaporation system. The evaporation performance of three different-sized AuNPs is studied in this report. All the evaporation rates of different-sized AuNPs saturated with the increase of the concentration due to the localization of the incident light at the water/air interface. We also found that under the same power input, even though small AuNPs exhibit no scattering effect, they absorb light and convert it to heat more efficiently than large-sized AuNPs with both light scattering function and light absorption function. To decrease the materials usage in the purely absorptive AuNP solution, we demonstrated that the introduction of purely scattering PSNPs not only had lowered the usage of AuNPs but also achieved higher evaporation efficiency via multi-scattering effect. Under the same mass density, the sizes of PSNPs must be optimized to maximize evaporation rate. Compared to the use of same NPs with both the scattering and absorptive properties^24^, this approach not only offers comparable performance, but also provides additional material flexibility in designing the absorptive centers and scattering centers. This approach should work as well for solid phase light-to-heat conversion systems with separated light absorbers and light scatters. The findings in this research also pave new way in optimizing the design of light-driven localized evaporation systems.

## Methods

### Preparation of metallic gold colloidal nanoparticles

#### Synthesis of 10-nm colloidal AuNPs^27^

Solution of 38.8 mM trisodium citrate dihydrate (99%, Aladin) was prepared by directly dissolving sodium citrate powder in DI water (NANOpure, Millipore Water Purification System; 18.2 MΩ). 1 mM tetrachloroauric acid (HAuCl_4_) (aq) was prepared by dissolving tetrachloroauric acid (49~50% Au basis; Aladin) in water. In a 100-mL flask, 50 mL of the HAuCl_4_ aqueous solution was heated to boiling with stirring. After quickly addition of 5 mL of 38.8 mM sodium citrate to the above solution, the solution was boiled for about 15 minutes and then cooled down to ambient temperature with stirring for additional 1 hour.

#### Synthesis of 50-nm and 100-nm colloidal AuNPs

Large colloidal AuNPs were synthesized by stepwise NH_2_OH-seeding method. Citrate capped 10-nm colloidal AuNPs were used as seeds to prepare 50-nm and 100-nm colloidal AuNPs. Stock aqueous solutions of 1% HAuCl_4_ and 0.294 M NH_2_OH were utilized as gold source and reducing reagent, respectively.

#### Preparation of concentrated 10-nm, 50-nm and 100-nm colloidal AuNPs

Concentrated 10-nm colloidal AuNP solutions were prepared by simply double the concentration of starting materials in a citrate reduction during the process represented above.

The newly made 100-nm colloidal AuNP solution was stored for two weeks. Due to nature sedimentation effects, 100-nm AuNPs precipitated at the bottom. The supernatant was removed and precipitates were remained at the bottom. They were collected and re-dispersed in DI water. Concentrated colloidal Au NP solutions were stored at room temperature.

Concentrated 50-nm AuNP solution was prepared in the same process. It, however, took a month for the sedimentation. All the experiments in this report used the same concentrated 10-nm, 50-nm, 100-nm colloidal AuNP solutions for evaporation test.

Note: AuNP solutions with different concentration used in this experiment are prepared by diluting the above as-prepared concentrated 10-nm, 50-nm and 100-nm AuNP solution. For example, the concentrated 10-nm AuNP serves as the original solution with a particle concentration of 2.298 × 10^13^ particles/ml, and the most diluted AuNP solution used in this experiment with a particle concentration of 0.359 × 10^12^ particles/ml was obtained by diluting the original concentrated AuNP solution by 64 folds with DI water.

#### Preparation of 200-nm PSNPs^32^

PSNPs with the size of 200 nm were synthesized according to soap-free emulsion polymerization method. 10 g of St (Styrene), 10 g of MAA (methacrylic acid), 0.24 g of NaHCO_3_ and 90 g of water were added into a 250-mL three-necked round-bottom flask attached with a stirrer, a reflux condenser, thermocouples and nitrogen gas inlet system. The solution was deoxygenated by bubbling nitrogen gas at room temperature for an hour. Next, the flask was immersed in a 78 °C water bath and vigorously stirred. After the addition of 0.2 g APS (Ammonium persulphate dissolved in 5 mL water), polymerization was triggered and the reaction lasted for 10 hours. The resulting colloidal PSNP suspension was purified by centrifugation at 12000 rpm to eliminate any large agglomerates for later use.

### Evaporation rate measurement under laser illumination

To measure the weight change of the water during the evaporation process, a 45-mL cuvette wrapped by aluminum foil and filled with AuNP solution sample was placed on a 4 decimal electronic precision balance (FR124CN, Ohaus Instrument, Shanghai). A green laser beam (VA-I-N-532, Bangshou Corp, Beijing) with a diameter of 3 mm was employed to perpendicularly illuminate the water/air interface. The evaporation weight change was instantaneously recorded as a function of time by a PC connected to the electronic balance.

### Determination of the mass of AuNPs in the solution

The data of mass of concentrated10-nm, 50-nm and 100-nm AuNPs are all collected directly from ICP-AES mass concentration measurement (Thermo Electron CORP, Model: iCAP 6000 Radial) with all AuNPs dissolved by moderate amount of aqua regia (the mixture of hydrochloric acid and nitric acid with a ratio of 3:1).

### Simulated value of the cross-section of large sized AuNPs and PSNPs

A FDTD (Finite Difference Time Domain) method is used to solve Maxwell equation to obtain cross section for homogeneous sphere. Values of the complex dielectric function of gold at different wavelengths were obtained directly from Johnson and Christy^33^, A refractive index value of 1.56 + 0i was used for polystyrene at all wavelengths, and the simulation medium is set as water.

### Lambert-Beer Law under the concentration studied in this report

For pure absorber 10-nm AuNPs, absorbance of different concentration from 1 fold to 30 fold was collected on a UV-Vis spectrometer (Ocean Optics, Model: HR2000 + CG) with a standard 10 mm × 10 mm × 45 mm cuvette. The absorbance **A** at 532 nm and the molar concentration of AuNPs (aq) **c** showed a linear relationship, expressed as A = 4.18 × 10^4^ c, which agrees well with the Lambert-Beer law.

### Free mean path length (*l*_*s*_)

For particles in a diluted solution, Lee *et al.* expressed the Lambert-Beer law for single scattering event as^34^:


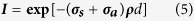


where ***I*** is the ratio of emerging light intensity to incident light intensity, 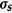 is scattering cross section of particles, 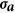 is absorption cross section of particles, 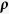 is particles concentration, ***d*** is path length of light. For purely scattering particles like PSNPs, 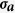 ≈ 0.

This equation can also be expressed as^35^:


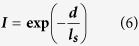


where 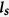 is the scattering free mean path length. By comparing the two equations above, for purely scattering particles solution, we consider the first order scattering and derive the expression of 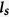 in terms of scattering cross section of particles and particles concentration:


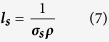


Under 532-nm laser light illumination, the simulated scattering cross sections of 200-nm PSNP and 500-nm PSNP are 3.01 ×**10^−15^** m^2^ and 1.65 × **10^−13^** m^2^ respectively.

## Additional Information

**How to cite this article**: Zhao, D. *et al.* Enhancing Localized Evaporation through Separated Light Absorbing Centers and Scattering Centers. *Sci. Rep.* **5**, 17276; doi: 10.1038/srep17276 (2015).

## Supplementary Material

Supplementary Information

## Figures and Tables

**Figure 1:**
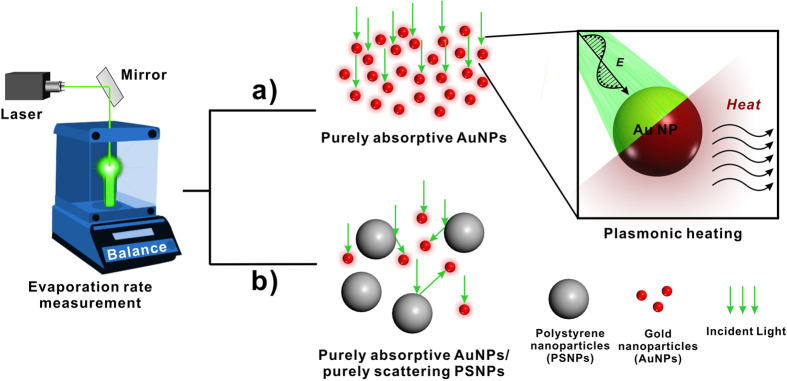
Schematic of evaporation performance set up of (**a**) purely absorptive aqueous AuNP solution and (**b**) purely absorptive AuNPs mixed with purely scattering PSNPs under 532-nm laser light illumination. The induced plasmonic heat is utilized to enhance the evaporation of water. (Fig. 1 was drawn by Chengyi Song).
