# Supplementary material for: Enhancing Localized Evaporation through Separated Light Absorbing Centers and Scattering Centers
Source: Sci Rep. 2015 Nov 26;5:17276. doi: 10.1038/srep17276 (PMC4660318; doi:10.1038/srep17276)
Supplement: Supplementary Information [file srep17276-s1.pdf]

Supporting information for:

Enhancing Localized Evaporation through Separated Light Absorbing Centers and Scattering Centers

Dengwu Zhao,[‡1] *Haoze Duan,*[‡1] *Shengtao Yu,*[1] *Yao Zhang,*[1] *Jiaqing He,*[1] *Xiaojun Quan,*[2] *Peng Tao,*[1] *Wen Shang,*[1] *Jianbo Wu,*[1] *Chengyi Song**[1], *and Tao Deng**[1]

[1]State Key Laboratory of Metal Matrix Composites, School of Materials Science and Engineering, Shanghai Jiao Tong University, 800 Dong Chuan Road, Shanghai 200240, P.R.China.

[2]MOE Key Laboratory for Power Machinery and Engineering, School of Mechanical Engineering, Shanghai Jiao Tong University, 800 Dong Chuan Road, Shanghai 200240, P.R.China.

*Corresponding author: chengyi2013@sjtu.edu.cn; dengtao@sjtu.edu.cn

‡D. Zhao and H. Duan equally contributed to this work

Supplementary Data

Sample		10 nm AuNPs (17.95×10^{12}/ml)	50 nm AuNPs (7.26×10^{10}/ml)	100 nm AuNPs (9.81×10^{9}/ml)
Steady-state Evaporation Rate (mg/s) for the most concentrated AuNPs solution	Trial 1	0.41454	0.29810	0.30420
	Trial 2	0.41247	0.29480	0.29600
	Trial 3	0.41696	0.32162	0.30920
Average *Steady-state Evaporation Rate* (mg/s)		0.41465	0.30484	0.30313
Percentage of change		0.54%	4.79%	2.20%

Table S1. Change of steady-state evaporation rate of concentrated solution of AuNPs with different sizes. (The three trials were the repeated measurements for the same sample solutions. The sample solutions were heated up by the laser, and the evaporation rates were recorded as the rate reached steady state. During the repeating experiments, the sample solutions were cooled down to room temperature, heated up by laser, and steady state evaporation rates were measured again.)

Reflectivity at the air-water interface:

For the 532-nm laser light used in the experiment, the calculated reflection at the air-water interface is ~2.1% based on the reflectivity calculation below (Grüniger H, et al. *The Journal of Chemical Physics*, **1983**, 79(8): 3701-3710.):

$$\boldsymbol{R} = \left(\frac{\boldsymbol{n_2} - \boldsymbol{n_1}}{\boldsymbol{n_2} + \boldsymbol{n_1}}\right)^2$$

where $\boldsymbol{R}$ is the reflectivity, $\boldsymbol{n_1}$ is the refractive index of air and $\boldsymbol{n_2}$ is the refractive index of water. The refractive index of water is 1.335, so for the pure water, this reflectivity at the air-water interface is 2.1%.

For the nanoparticle solutions, a Maxwell Garnett approximation is used to calculate the dielectric constant (See the calculation below). In the nanoparticle solutions, the volume fraction of either AuNPs or PSNPs used in this experiment is relatively small, so the calculated refractive indexes are all close to that of pure water (~1.335). **Table S2** shows the calculated dielectric constants and refractive indexes for the solutions containing only 10-nm particles, and the calculated results for solutions containing 50-nm and 100-nm particles are also similar. Due to these similar refractive indexes for the solution, the reflection at the air-water interface is thus all close to 2%.

Maxwell Garnett approximation equation (Salvatore Torquato. '*Random Heterogeneous Materials'*. Springer New York. **2002**. Chapter 18):

$$\frac{\boldsymbol{\sigma_e} - \boldsymbol{\sigma_1}}{\boldsymbol{\sigma_e} + \boldsymbol{2\sigma_1}} = \sum_{j=1}^{M} \boldsymbol{\rho_j}\left[\frac{\boldsymbol{\sigma_j} - \boldsymbol{\sigma_1}}{\boldsymbol{\sigma_j} + \boldsymbol{2\sigma_1}}\right]$$

Where $\boldsymbol{\sigma_e}$ is effective dielectric constant, $\boldsymbol{\sigma_1}$ is dielectric constant for medium 1, $\boldsymbol{\rho_j}$ is volume fraction of medium $\boldsymbol{j}$, $\boldsymbol{\sigma_j}$ is dielectric constant for medium $\boldsymbol{j}$. The results are shown in **Table S2**.

Sample	Dielectric constant	Refractive index	Reflection
10nm AuNPs<br>($\mathbf{0.359} \times \mathbf{10^{12}/ml}$)	$\mathbf{1.78223}$<br>$\mathbf{+1.73637 \times 10^{-6} i}$	$\mathbf{1.3350}$<br>$\mathbf{+6.503 \times 10^{-7} i}$	**2.1%**
10nm AuNPs<br>($\mathbf{1.795} \times \mathbf{10^{13}/ml}$)	$\mathbf{1.78231}$<br>$\mathbf{+2.809 \times 10^{-6} i}$	$\mathbf{1.3350}$<br>$\mathbf{+1.052 \times 10^{-6} i}$	**2.1%**
10nm AuNPs<br>($\mathbf{0.359 \times 10^{13}/ml}$)<br>And<br>200nm PSNPs<br>($\mathbf{0.607 \times 10^{11}/ml}$)	$\mathbf{1.78237}$<br>$\mathbf{+1.73648 \times 10^{-6} i}$	$\mathbf{1.3351}$<br>$\mathbf{+6.503 \times 10^{-7} i}$	**2.1%**
10nm AuNPs<br>($\mathbf{0.359 \times 10^{13}/ml}$)<br>And<br>200nm PSNPs<br>($\mathbf{3.04 \times 10^{11}/ml}$)	$\mathbf{1.78296}$<br>$\mathbf{+1.73685 \times 10^{-6} i}$	$\mathbf{1.3353}$<br>$\mathbf{+6.504 \times 10^{-7} i}$	**2.1%**

Table S2. Effective medium model calculation results for different aqueous nanoparticle solutions.

Total reflection and backscattering

The total reflection from the solution consists of reflection at the air-water interface (as calculated above) and also the reflection due to the back scattering from the particles dispersed in the solution. In our model, we defined $\mathbf{d}_s$ as the thickness of the top layer of solution that is responsible for the back scattering reflection, and its values were set based on the analysis of the images of the solution under light illumination. The back scattering reflectivity is equal to $I_{back}/I_{incident}$ (I_{back} is back scattering light intensity and $I_{incident}$ is incident light intensity). We calculated the ratio of I_{back} to total scattering light intensity ($I_{back}/I_{scatter}$) by using FDTD simulation. The total-field scattered-field source was used in the FDTD simulation, and the scattering intensity distributions of the nanoparticles were computed analytically through the use of many monitor boxes. Based on the calculated reflection at the air-water interface and also the back scattering from the particles, the total reflectivities of different nanoparticle solutions were calculated and plotted versus nanoparticle concentration ρ in **Figure S1**. As shown in Figure S1b, as the concentration of 50-nm AuNP increases, there will be more optical energy lost due to the total reflection back out of the air-water interface, and that loss helps explain the saturation (**Figure 3b**). For 100-nm AuNP, the increased reflection at higher concentration (**Figure S1c**) can also help explain the trend of saturation of evaporation rate (**Figure 3c**). For mixed solutions, we took 10-nm AuNP/200-nm PSNP as an example, and the increased reflection at higher concentration of PSNP (**Figure S1d**) also possibly led to the decrease in the rate enhancement observed in **Figure 4b**.

Penetration depth:

Penetration depth (defined as the path length through which incident light is attenuated by 99%) is calculated according to Lambert-Beer Law as follow (see the Ref.28 in the manuscript):

$$d_a = \frac{ln\, 99}{(\sigma_a + \sigma_s)\rho}$$

where d_a is the penetration depth, σ_a is the absorption cross section and σ_s is scattering cross section (FDTD is used again to calculate the scattering cross section and absorption cross section of all the nanoparticles including different sized AuNPs and PSNPs in this experiment.), ρ is nanoparticle concentration. The penetration depths, thermally play key roles in defining the lengths of hot zones observed in the IR images, of different nanoparticle solutions are also plotted versus nanoparticle concentration ρ in **Figure S1**.

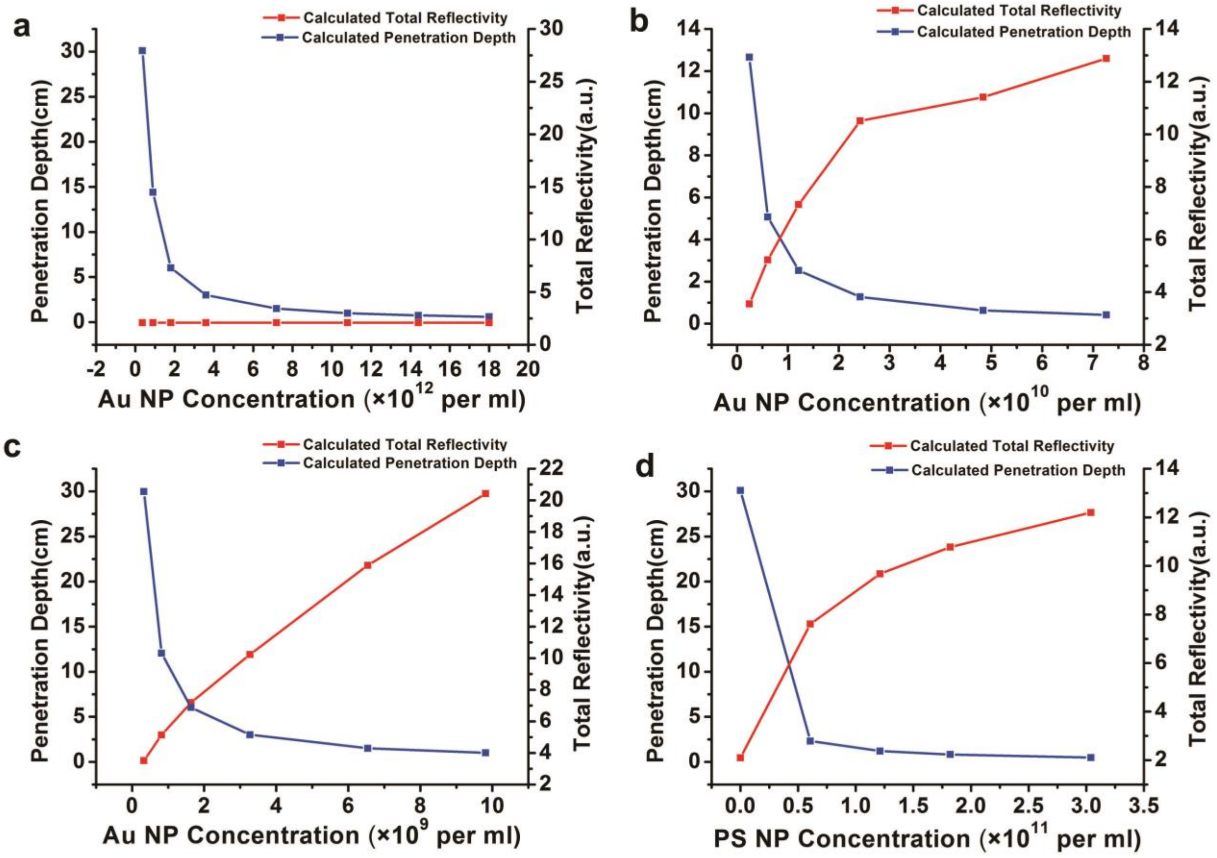

Figure S1. Change of calculated penetration depth and calculated total reflectivity with different particle concentration. a) Pure 10-nm AuNP (aq); b) Pure 50-nm AuNP (aq); c) Pure 100-nm AuNP (aq); d) Mixed solution of 10-nm AuNP and 200-nm PSNP (aq) with fixed concentration (0.359×10^{12}/ml) of 10-nm AuNP.

Absorbed power at the evaporation interface:

To evaluate the evaporation performance of different nanoparticle solutions, the modeling will be more complex, and it will involve both optical and thermal modeling. Here we took a simplified approach that focuses on optical absorption to try to compare the trend observed. In the following, we assume that the absorbed light power density $\boldsymbol{P}$ within the top layer of solution with thickness $\mathbf{d}_{\boldsymbol{e}}$ is responsible for surface water evaporation. In our calculation, $\mathbf{d}_{\boldsymbol{e}}$ is set as 1cm for all experimental samples, which is approximately the shortest hot zone length analyzed from IR images. Assuming that the incident light excluding the total reflected light is all absorbed, the power density $\boldsymbol{P}$ is thus calculated by the following equation:

$$\boldsymbol{P} = \frac{\boldsymbol{P_{incident}} \times (\mathbf{1} - \boldsymbol{R_{total}}) \times \ (\mathbf{1} - \boldsymbol{e}^{-\boldsymbol{\rho}\times\boldsymbol{\sigma_e}\times\boldsymbol{d_e}})}{\boldsymbol{d_e} \times \boldsymbol{S}}$$

where $\boldsymbol{P_{incident}}$ is total incident power, $\boldsymbol{R_{total}}$ is calculated total reflectivity above, $\boldsymbol{\rho}$ is the nanoparticle concentration, $\boldsymbol{S}$ is the cross section of solution sample, and $\boldsymbol{\sigma_e}$ is the effective nanoparticle extinction cross section, which includes the extinction contribution from both the AuNPs and PSNPs. The calculated power density $\boldsymbol{P}$ of different nanoparticle solutions is plotted versus nanoparticle concentration $\boldsymbol{\rho}$ in **Figure S2**.

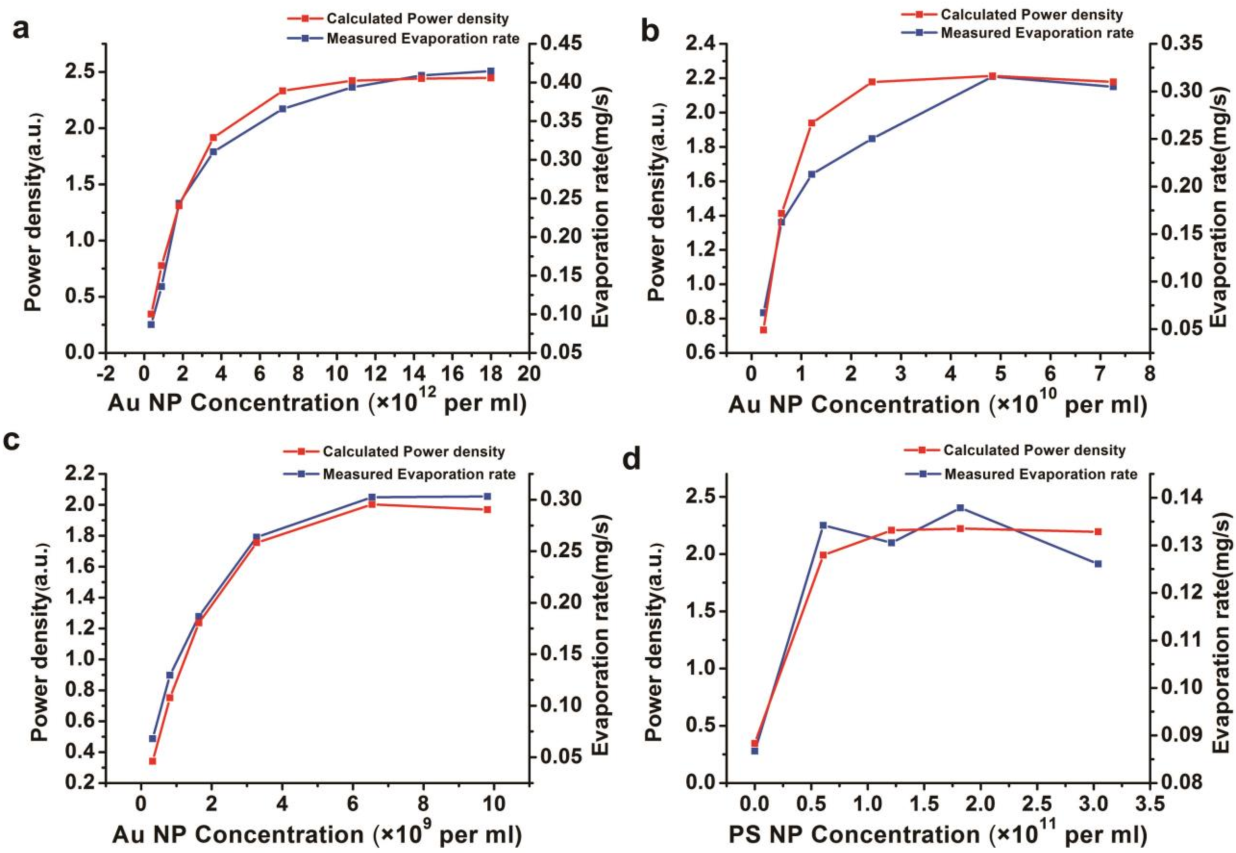

Figure S2. Change of calculated power density and measured evaporation rate with different particle concentration. a) Pure 10-nm AuNP (aq); b) Pure 50-nm AuNP (aq); c) Pure 100-nm AuNP (aq); d) Mixed solution of 10-nm AuNP and 200-nm PSNP (aq) with fixed concentration (0.359×10^{12}/ml) of 10-nm AuNP.
